# Combination of nitrogen and organic fertilizer practices increased rice yields and quality with lower CH_4_ emissions in a subtropical rice cropping system

**DOI:** 10.3389/fpls.2025.1613163

**Published:** 2025-05-21

**Authors:** Hongbao Wu, Yakang Jin, Yongjie Qi, Ruilin Huang, Fuwei Wang

**Affiliations:** College of Resources and Environment, Anhui Science and Technology University, Chuzhou, China

**Keywords:** CH_4_ emission, rice grain yield, nutritional quality, nitrogen application rate, organic fertilizer

## Abstract

Fertilizer nitrogen (N) application has been shown to impact methane (CH_4_) emissions, yield and quality from rice cropping systems, yet the responses of CH_4_ fluxes, yield and quality to N reduction and combined application of organic fertilizer in subtropical rice cropping systems are not well documented. Six experimental treatments were conducted: N90 kg N ha^-1^ of urea (N1), organic fertilizer with equal N90 (O1) and 80% urea + 20% organic fertilizer (N1O1), farmer’s common practice with N270 kg N ha^-1^ of urea (N2), organic fertilizer with equal N270 (O2) and 80% urea + 20% organic fertilizer (N2O2) were conducted to simultaneously measure the CH_4_ flux, yield and quality from a subtropical rice cropping system in south China. Results showed that increased N fertilizer application significantly stimulated soil CH_4_ emission, increased rice yield and altered quality in paddy fields. CH_4_ emissions were quantified under different N fertilizer management practices in the peak rice growing season during the tillering and heading stages, respectively. Organic fertilizer alone increased CH_4_ emission by 442.1% in O1 and by 337.3% in O2 compared with urea. However, relative to organic fertilizer, organic fertilizer combined with urea significantly decreased CH_4_ emissions by 48.4% in O1 and by 39.2% in O2. Compared with N1 and N2 treatment, rice yield was significantly decreased by 34.4% and 39.5% under O1 and O2, while significantly enhanced by 49.8% and 22.3%, respectively, under N1O1 and N2O2 (*P* < 0.05). The protein content significantly increased under N1O1 by 18.8% and 41.5%, the amylose content by 30.3% and 14.8%, and the gel consistency by 32.7% and 15.5% in contrast to N1 and O1 (*P* < 0.05). Similarly, the protein content, amylose content and gel consistency under N2O2 were consistent with the rice quality under the N1O1 treatments above. In summary, optimizing organic fertilizer combined with urea practices was a win-win strategy to improve grain yield and quality while reducing CH_4_ emissions in the rice cropping system. This study provides new insights into the fertilizer types on CH4 emission and rice production of rice cropping systems.

## 1 Introduction

At present, global warming has become an indisputable fact among governments and academic circles. Methane (CH_4_), the second most important anthropogenic greenhouse gas (GHG) aftercarbon dioxide (CO_2_), has a global warming potential 28 times that of CO_2_ over 100 years and contributes about 20% to global warming, increasing at 1% per year (Dlugokencky et al., 2011; Wu et al., 2020). Agricultural soils account for approximately 50% of global anthropogenic CH_4_ emissions (Zhou et al., 2015; Zhang et al., 2019), particularly in rice cropping systems (Li et al., 2018; Lou et al., 2024). Rice cultivation is the major anthropogenic source of atmospheric CH_4_, with 20-40 Tg y^−1^ being released from rice fields, contributing approximately 11%-17.9% of the total global CH_4_ emissions (Sass et al., 1999; Shi et al., 2010; Montzka et al., 2011; Li et al., 2018; Kim et al., 2016). Therefore, in order to reduce atmospheric CH_4_ emissions and mitigate global warming, numerous previous studies have focused on management practices to decrease CH_4_ emissions from rice fields, especially regarding N fertilizer application (Li et al., 2018).

N fertilizer input is an essential factor to optimize and increase rice yield (Li et al., 2018; Zhang et al., 2020; Hu et al., 2021), with a contribution rate of 50% to increase production (Wu and Wang, 2002). China is the largest global rice producer, accounting for approximately 28% of the cultivation area with 30.14×10^6^ hm^2^ and 28.1% of global rice production, which feeds 22% of the world population (Liang et al., 2017; Wu et al., 2018; Li et al., 2018). Meanwhile, the grain crop N use efficiency of only 30%-40% in China is considerably lower than the world average of 50% (Zhu, 2000; Zhang et al., 2008), which generates a cascade of environmental issues, e.g. water eutrophication, soil acidification and CH_4_ emissions (Chen et al., 2014; Wang et al., 2014; Li et al., 2018). It is estimated that CH_4_ emissions from rice paddies in China account for approximately 25% of the world’s rice cropping systems (Wu et al., 2018). In consideration of the importance of the N fertilization effect on CH_4_ emissions in rice fields, numerous studies have been conducted (Tang et al., 2017; Wu et al., 2018; Li et al., 2018). However, the influence of N fertilizer inputs on CH_4_ emission in rice fields remains uncertain, with previous studies reporting increases (Tang et al., 2017; Liu et al., 2019; Cao et al., 2022), or decreases (Ji et al., 2014; Yao et al., 2012; Miao et al., 2020). Previous studies with an increasing view suggested that N fertilizer addition was the key factor of CH_4_ emission in paddy fields, which can significantly increase CH_4_ emission fluxes, in particular, organic fertilizer application (Wu et al., 2018; Liu et al., 2019; Miao et al., 2020). The application of N fertilizer in paddy soil will significantly increase the soil available nitrogen content, which will provide abundant nitrogen sources for methanogens (Wu et al., 2020; Jin et al., 2024). Meanwhile, N application can increase the biomass of rice plants and roots, which biomass litter decomposition and root exudation provide more organic substrate availability for methanogens (Jia et al., 2001; Kerdchoechuen, 2005; Kim et al., 2016; Zhang et al., 2019). Moreover, the application of organic fertilizer can significantly soil microbe populations and enzyme activities, while simultaneously increasing the soil humus content and exogenous carbon, which will provide better conditions for CH_4_ production in paddy soil and subsequently stimulate methane production CH_4_ emissions (Tang et al., 2017; Wu et al., 2018; Jin et al., 2024). Recent previous studies, in contrast, demonstrated that N fertilization can significantly decrease CH_4_ emissions by 14%-50% in rice-based cropping systems (Ji et al., 2014; Yao et al., 2012; Zhou et al., 2015; Miao et al., 2020). N fertilization could stimulate rhizosphere development in rice and subsequently improve root oxygen transport in the extremely reduced soil, stimulate methanotrophs growth, as well as increase CH_4_ consumption through enhanced methanotrophic microbial activities (Yao et al., 2012; Hu and Lu, 2015; Zhou et al., 2015; Kim et al., 2016).

The above conflicting results may be due to the limitation of monitoring point selection (Kim et al., 2016) or applied N fertilizer too deeply into the soil (Chen et al., 1993), but we are more convinced that variation in soil methanogens and methanotrophs resulted in this conflicts because the net CH_4_ emissions in rice-based cropping systems were controlled from the balance between the CH_4_ produced by methanogens and oxidized by methanotrophs (Hu and Lu, 2015; Li et al., 2018; Liu et al., 2019). Additionally, CH_4_ emission in rice fields is also the result of complex interactions between rice plants and soil microorganisms (methanogens and methanotrophs), which the result is influenced by competition for available N in soil between microorganisms and rice plants (Liu et al., 2019; Li et al., 2018; Zhang et al., 2019). However, the above studies only discussed the effects of N fertilizer (urea or organic fertilizer) on CH_4_ emissions in rice soil, and did not further analyze how the changes in CH_4_ production and consumption genes affected CH_4_ flux under N fertilizer management measures. Most previous studies dealing with CH_4_ emissions in paddy soils affected by N fertilization have mostly considered only a single factor (e.g., either urea or organic fertilizer) (Zhou et al., 2015; Kim et al., 2016; Cao et al., 2022), and there are limited data available regarding the interactions among these factors. Although a few studies on the effects of nitrogen fertilizer application on methanogens and methanogens microbial communities have been reported, no consensus has been achieved (Schimel, 2000; Yao et al., 2012; Hu and Lu, 2015; Liu et al., 2019). In particular, the response of CH_4_ emissions to organic fertilizer mixed with N fertilizer and the underlying mechanisms remain unclear. Thus, to clarify the response of CH_4_ under different N fertilizer management, we investigated the rice cropping systems in southern China.

Apart from the impact on CH_4_ emissions, N application had an obvious regulation effect on rice yield and quality (Gu et al., 2015; Li et al., 2018; Zhang et al., 2020; Hu et al., 2021). The net photosynthetic rate and chlorophyll content of plants were significantly increased by suitable N fertilizer application, which promoted rapid growth of rice, accounting for approximately 50% of increased grain production (Wu and Wang, 2002; Zhang et al., 2020). The results reported by Wu et al. (2018) showed that chemical fertilizer, pig manure+chemical fertilizer, chicken manure+chemical fertilizer and rice straw+chemical fertilizer significantly increased rice yield by 18.1%, 30.1%, 38.9% and 35.1%, respectively, compared with no N fertilizer. Meanwhile, in order to improve N use efficiency, a 25% N reduction significantly increased rice yield by 32.5% (Tang et al., 2020). Numerous previous studies have demonstrated that optimizing N fertilizer management is also an important measure to improve the quality of rice, which significantly increases the head rice rate, high viscosity and breakdown values, protein content and decreased amylose content (Ju et al., 2018; Tang et al., 2020; Hu et al., 2021). Nevertheless, excessive N fertilization significantly increased the brown rice rate, chalkiness and significantly decreased amylose content, worsening rice appearance, cooking, and eating quality (Li et al., 2024; Tang et al., 2020; Hu et al., 2021). Tang et al. (2020) suggested that optimizing N fertilizer management increased the protein content and the head rice rate by 29.7% and 20.0%, respectively, while decreasing the amylose content by 28.8%, compared with no N fertilization.

Numerous past studies have only considered the effects of N application on CH_4_ emissions in paddy fields or on rice quality and yield, while did not involve comprehensive studies on environmental problems such as CH_4_ emissions in paddy fields caused by N application under the premise of ensuring rice quality and yield. Therefore, it is timely to investigate the effects of organic and inorganic combined application on CH_4_ emission and yield and quality under different N application levels, in particular, the microbiological mechanism of CH_4_ production. This study aimed to evaluate the combined impacts of organic and inorganic combined application under different N fertilizer levels on CH_4_ emissions and rice yield and quality and to investigate the underlying mechanisms on controlling CH_4_ emissions from rice production systems when the above N fertilizer management conditions.

## 2 Materials and methods

### 2.1 Site description

The field experiment was conducted in a paddy field on campus farms at Anhui Science and Technology University in Chuzhou City, Anhui Province, China (32°87′N, 117°56′E) in 2023. The experimental area is in a subtropical humid monsoon climate zone with a mean annual temperature of 14.9°C and total precipitation of 904.4 mm. This area of China is typically recognized for the rice-wheat rotation systems in which the wheat growing season is from November of the previous year to June of the following year and the rice growing season from June to November. This experimental site was established in 2018, where fertilization experiments have been conducted with rice-wheat rotations for six years. In the paddy field, the clay soil had the following nutrient contents in 2023, respectively: 8.05 pH, 0.49 g·kg^-1^ total N, 63.52 mg·kg^-1^ alkali-hydrolyzable N, 0.51 g·kg^-1^total phosphorus, 3.83 mg·kg^-1^ rapidly available phosphorus, and 13.51 g·kg^-1^ organic matter.

### 2.2 Experiment design

The experiment included six treatments: low N (90 kg N ha^-1^) as urea (N1), organic fertilizer (O1), and 80% N from organic + 20% N from urea (N1O1); and high N (270 kg N ha^-1^) as urea (N2), organic (O2), and 80% N from organic + 20% N from urea (N2O2) (**Table 1**). The plots were arranged in a randomized complete block experimental design. Each treatment had three replicates (18 plots in total), each plot was 3.75 m^2^. To avoid interference through the exchange of water and fertilizer, each plot was isolated using concrete bricks.

**Table 1:** Experimental design with two factors, i.e., organic fertilizer and N fertilizer rate.

Level	Treatment	Factors	
		organic fertilizer rate (kg N ha^-1^)	N fertilizer rate (kg N ha^-1^)
LN	N1	0	90
	O1	90	0
	N1O1	18	72
HN	N2	0	270
	O2	270	0
	N2O2	54	216

The field trial was conducted from July 1 to October 15, 2023, using rice cultivar Yangdao 6 transplanted at 19 plants·m^-2^. Uniform phosphorus and potassium applications were maintained across all treatments: calcium superphosphate (12% P_2_O_5_) at 75 kg·ha⁻¹ and potassium sulfate (60% K_2_O) at 150 kg·ha⁻¹ annually. Organic fertilizer, P and K were applied basally, while urea was split: 50% basal, 30% tillering, 20% grain-filling.

### 2.3 CH_4_ sampling and measurement

CH_4_ samples were collected at the early stage of rice growth using static closed chambers with length 50 cm, width 50 cm and height 50 cm, while at the late stage of rice growth with length 100 cm, width 50 cm and height 50 cm, respectively, from 08:00-11:00 a.m. Four corrosion-resistant steel enclosures were embedded 10 cm below the soil surface seven days preceding initial sampling, remaining installed throughout the study. Each chamber’s exterior received dual thermal regulation: rubber foam insulation coupled with reflective aluminum cladding to reduce radiative heat transfer during measurements. A 60 mL syringe facilitated timed gas extraction, capturing 40 mL chamber headspace at four intervals (closure initiation, +5, +15, and +30 min) with concurrent temperature monitoring. Acquired samples underwent immediate transfer to 12 mL vacuum-sealed containers, followed by < 24 h laboratory analysis via GC system (Agilent 7890, USA).

### 2.4 CH_4_ flux calculation

The calculation of CH_4_ flux and its cumulative emission flux were described by Jin et al. (2024).

### 2.5 Soil sampling and measurements

At rice physiological maturity, composite soil cores (0–20 cm depth) were obtained from experimental plots for physicochemical characterization. Three replicates surrounding each plot were well blended thoroughly as one sample. Repeat sampling 3 times for each of the above mixed soil samples. The samples used for the determination of soil nutrients were dried at room temperature, crushed, and sieved to pass through a 2 mm mesh. Another samples used for the determination of methyl-coenzyme M reductase alpha subunit (*mcrA*) and methane monooxygenase alpha subunit (*pmoA*) were frozen and stored at -80°C for subsequent determination and analysis. Elemental composition (C, N) quantification employed combustion analysis via a CHNS elemental analyzer (Elementar Vario EI, Germany). Mineral nitrogen speciation (NH_4_^+^-N and NO_3_⁻-N) determinations utilized continuous flow analysis technology (Skalar San++ System, Netherlands).

### 2.6 Quantitative PCR of *mcrA* and *pmoA* genes in extracted soil microbiome DNA

To quantify functional bacteria, genes of *mcrA* and *pmoA* were used as molecular markers to determine the copies of the above functional bacteria in rice soils during the harvest period. Quantitative PCR used the SYBR Green method with two primer pairs for *mcrA* and *pmoA* (**Table 2**).

**Table 2:** Amplification primers of *mcrA* and *pmoA*.

Primer	Microorganism functional genes	Specific primer sequences
MLfF_MLrR	*mcrA*	GGTGGTGTMGGATTCACACARTAYGCWACAGC
		TTCATTGCRTAGTTWGGRTAGTT
A189F_mb661R	*pmoA*	GGNGACTGGGACTTCTGG
		CCGGMGCAACGTCYTTACC

### 2.7 Rice sampling and measurements

The determination method of effective panicle number, grains per spike, thousand-grain-weight, theoretical yields and quality (protein, gel consistency and amylose) was described by Jin et al. (2024). Theoretical yield were calculated according to (Jin et al., 2024), using Equation 1:


(1) $$Y=P_{n}\times G_{n}\times TKW\times 85\%\times 667\times 15$$

where, *Y* is the theoretical wheat yield (kg hm^-2^), *P*n is the spikes per hectare, *G*n is the grains per spike, TKW is the thousand kernel weight”.

### 2.8 Statistical analysis

Statistical evaluations were conducted using IBM SPSS Statistics version 19.0 (IBM, Armonk, New York, NY, USA) and R software. The averages and standard errors were computed for both CH_4_ flux and the associated environmental variables. A one-way ANOVA was employed to assess the significance of the observed data. Statistical significance was considered at *P* < 0.05. Additionally, both linear and nonlinear regression analyses were utilized to explore the relationships between CH_4_ flux and environmental factors. Random forest models assessed the relationships between soil nutrient and CH_4_ emission and functional gene using rfPermute package.

## 3 Results

### 3.1 Effects of different N fertilizer practices on soil physicochemical properties

**Figure 1:**
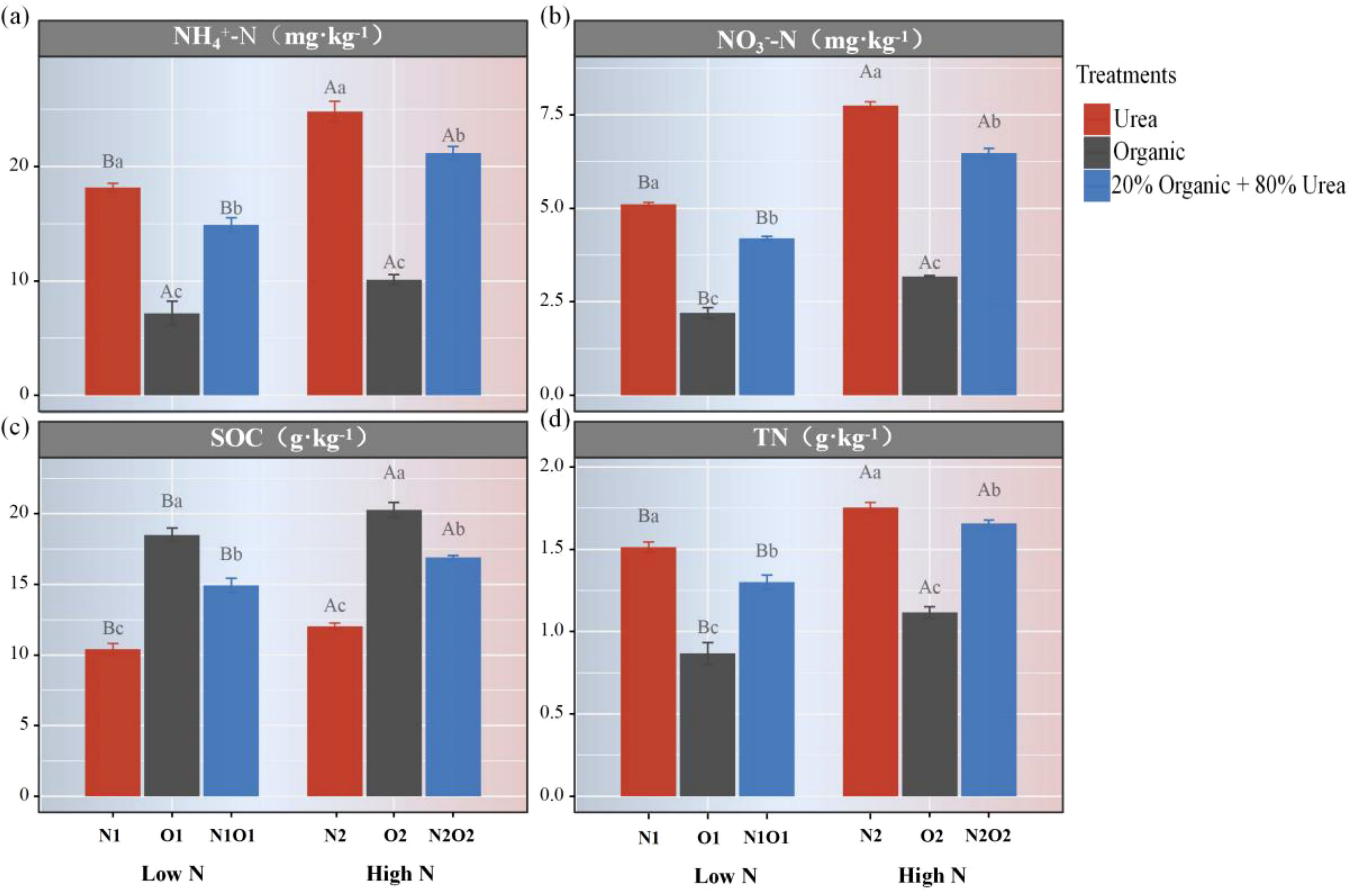
Differences in soil physicochemical properties among different treatments. Ammonium nitrogen content **(a)**, nitrate nitrogen content **(b)**, soil organic carbon content **(c)** and total nitrogen content **(d)**. Different lowercase letters indicated significant differences among treatments under the same N fertilizer application rate. Different capital letters indicated significant differences among the same treatments under different N application rates (P < 0.05). The same below.

To evaluate the impacts of the application rate and type of N fertilizer on soil physicochemical properties, NH_4_^+^-N, NO_3_⁻-N, SOC and TN were measured. Except for NH_4_^+^-N in O1 and O2, the content of NH_4_^+^-N, NO_3_⁻-N, SOC and TN in soil were significantly increased with the N application rate. Under equivalent nitrogen application rates, urea application exhibited significantly higher NH_4_^+^-N, NO_3_⁻-N and TN in soil (*P* < 0.05, **Figure 1**). In contrast, compared with urea, organic fertilizer alone significantly decreased the content of NH_4_^+^-N, NO_3_⁻-N and TN, while increased SOC content. Specifically, mean soil SOC content significantly increased by 77.6% and 68.9% in O1 and O2, respectively, relative to N1 and N2 (*P* < 0.05, **Figure 1**). Compared with urea or organic fertilizer alone, all measured parameters under organic fertilizer combined with urea fell between those of the above two treatments. These results suggest that organic fertilization may enhance the potential fertility of paddy soils but significantly reduce available nitrogen content during the current growing season.

### 3.2 Responses of methanogens and methanotrophs functional genes to N fertilizers practices

**Figure 2:**
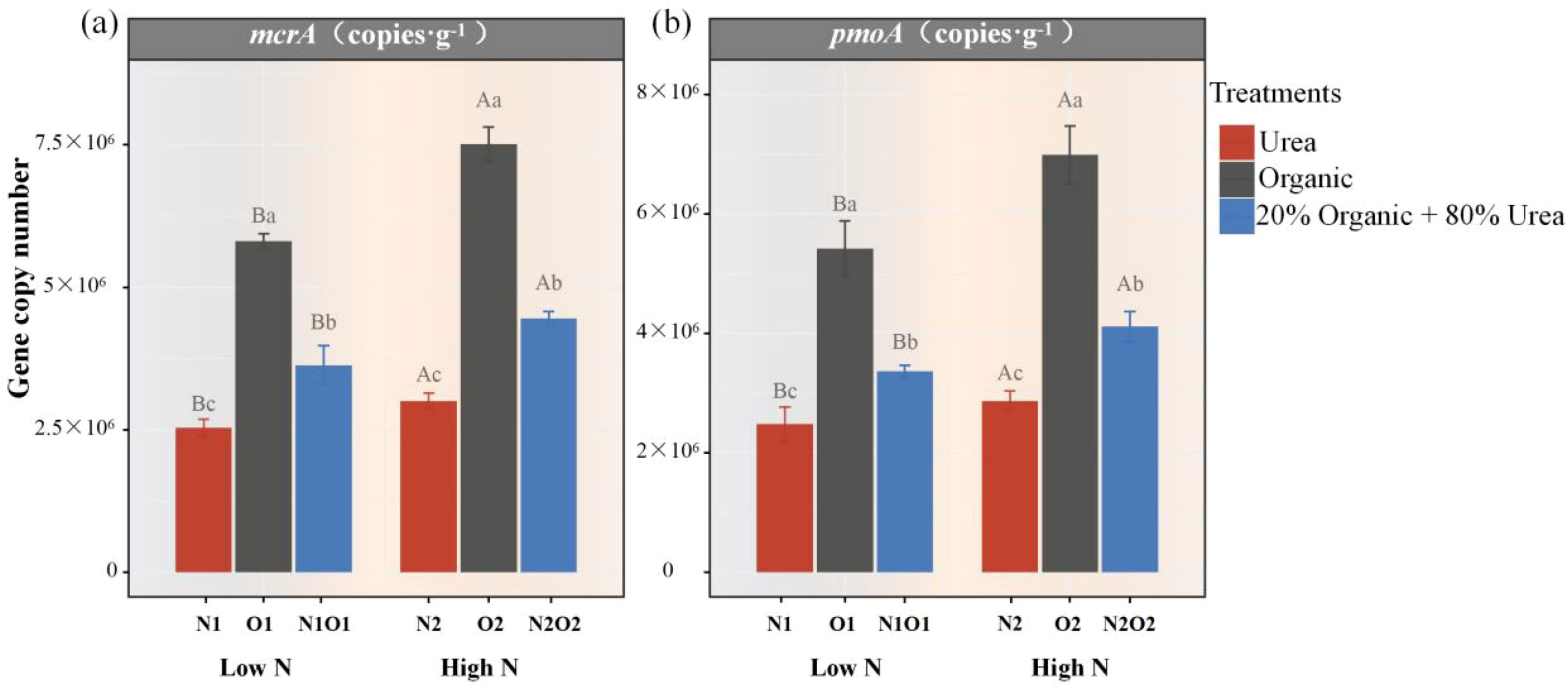
Average copy numbers of mcrA **(a)** and pmoA **(b)** under different N fertilizers practices.

To investigate the effects of N fertilizer practices on methanogens and methanotrophs potentials, the copy numbers of *mcrA* and *pmoA* were quantified via qPCR. Under low-N application rate, O1 significantly increased *mcrA* gene copies by 128.6% and 59.8% compared to N1 and N1O1, respectively (*P* < 0.05, **Figure 2a**). Under high-N application, mean *mcrA* gene copies were 7.51×10^6^ copies g^-1^ dry soil in O2, significantly higher by 149.9% and 68.7% than N2 and N2O2, respectively (*P* < 0.05). In addition, increased N application rates significantly enhanced *mcrA* gene copies under both low and high nitrogen conditions (*P* < 0.05). The response of pmoA to different N fertilizer practices was consistent with the trend of mcrA (*P* < 0.05, **Figure 2b**).

### 3.3 Responses of CH_4_ emissions to N fertilizers practices

**Figure 3:**
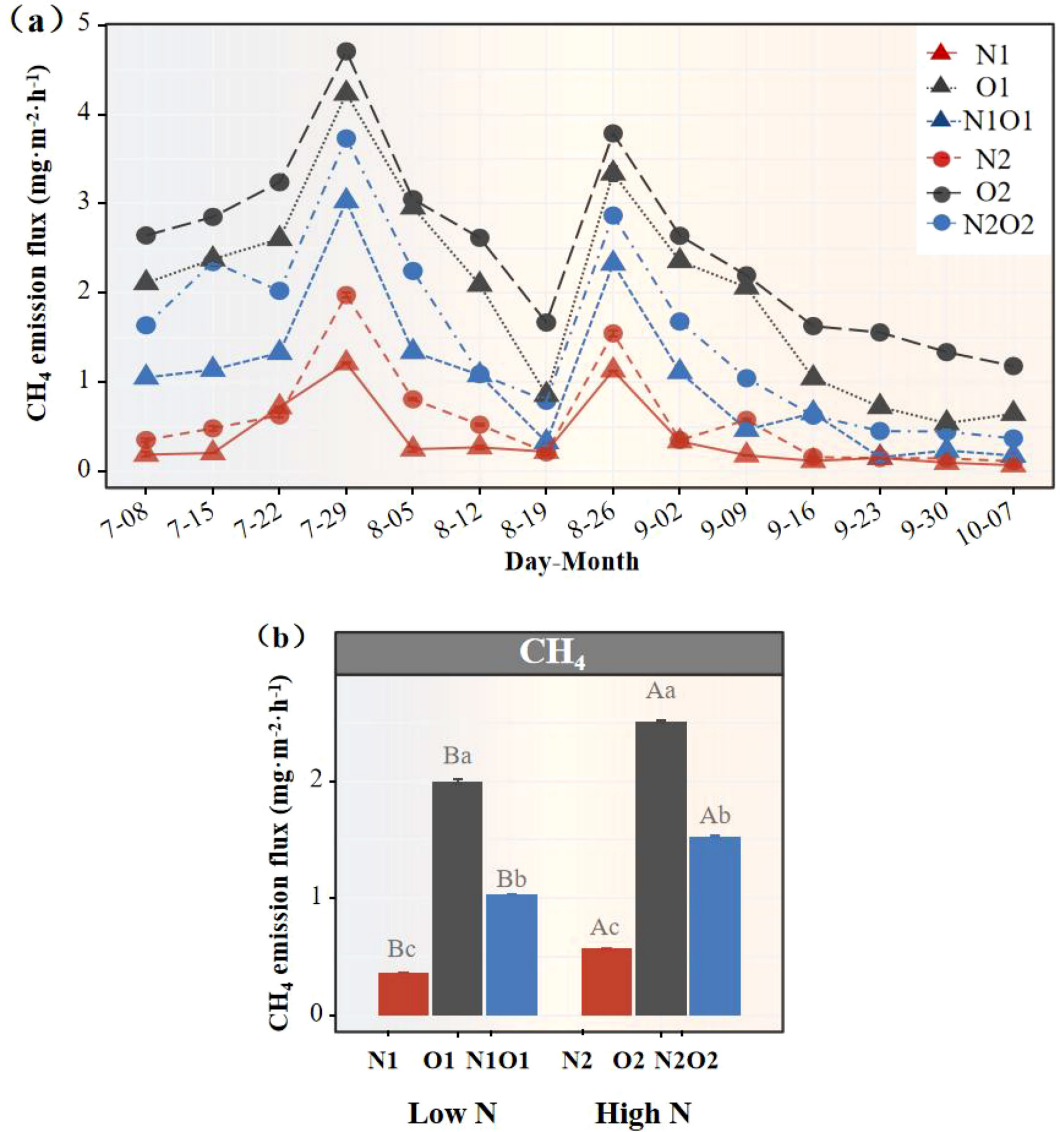
Variations in CH_4_ emissions under N fertilizers practices. Temporal changes in CH_4_ emissions under different N fertilizer treatments during the sampling periods **(a)**; Differences in a mean of CH_4_ emissions among treatments under N fertilizers practices **(b)**.

During the whole rice growing season, CH_4_ emissions ranged from 0.18 to 4.71 (mean of 1.33 ± 0.12) mg m^-2^ h^-1^, implying that the rice field soil was a net carbon source for atmosphere (**Figure 3a**). From July 8 to August 19, 2023, CH_4_ emissions from paddy fields generally followed the first rising and then decreasing, and then to the harvest period, the variation trend of CH_4_ emissions was the same as the above, with two peaks occurring at the tillering and heading stages. Further analysis of CH_4_ emissions revealed that under low-N application, O1 increased CH_4_ emissions by 4.42-fold compared to N1 and by 48.3% compared to N1O1 (*P* < 0.05, **Figure 3b**). Under high-N application, O2 resulted in 3.37-fold and 64.4% higher CH_4_ emissions than N2 and N2O2, respectively. Meanwhile, increased N fertilizer application also significantly increased CH_4_ emissions (*P* < 0.05, **Figure 3b**). In addition, increased N fertilizer application significantly stimulated soil CH_4_ emission (*P* < 0.05, **Figure 3b**).

### 3.4 Relationships between soil physicochemical properties and methanogenic functional genes

**Figure 4:**
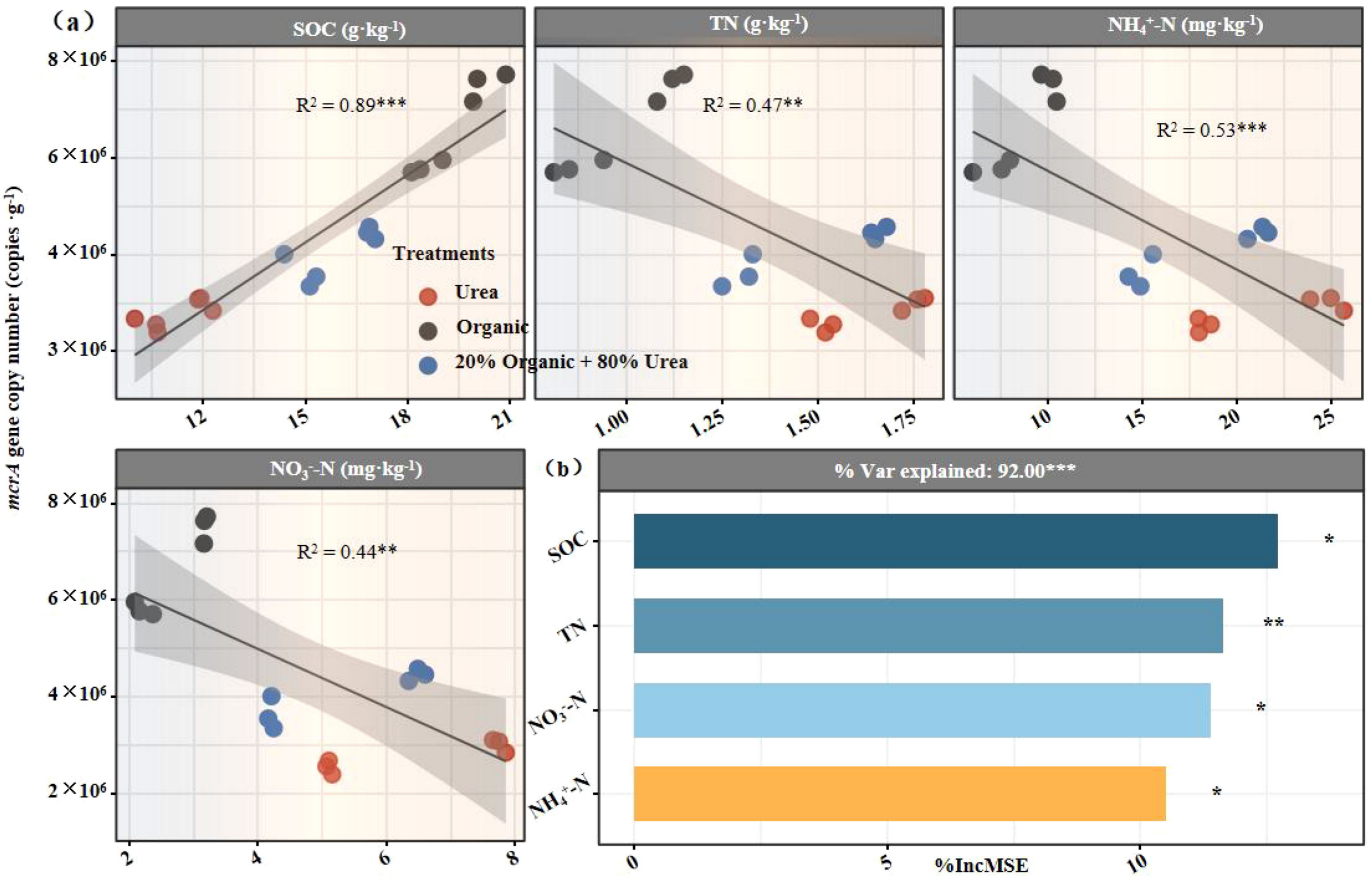
Relationships between soil physicochemical properties and methanogenic functional genes. Linear fitting analysis between soil physicochemical properties and methanogenic functional genes **(a)**; Random Forest analysis evaluating the importance of soil physicochemical properties to methanogenic functional genes **(b)**. *R* ^2^ represents goodness of fit, and asterisks (*) denote significance levels: * *P* < 0.05, ** *P* < 0.01, *** *P* < 0.001.

Linear regression and random forest analyses were employed to explore associations between soil properties and *mcrA* gene copies. Linear regression showed that SOC was significantly positively correlated with *mcrA* gene copies (*R*² = 0.89, *P* < 0.001; **Figure 4a**). In contrast, TN (*R*² = 0.47, *P* < 0.001), nitrate nitrogen (*R*² = 0.44, *P* < 0.001), and ammonium nitrogen (*R*² = 0.53, *P* < 0.001) were significantly negatively correlated with *mcrA* gene copies. The random forest model incorporating SOC, pH, nitrate nitrogen, ammonium nitrogen, and TN explained 93.2% of the variation in *mcrA* gene copies (*P* < 0.001; **Figure 4b**), with SOC identified as the most influential factor. These results suggest that the elevated SOC content induced by organic fertilization was the primary driver of increased methanogenic potential and methane emissions.

### 3.5 Relationship between CH_4_ emissions and environmental factors

**Figure 5:**
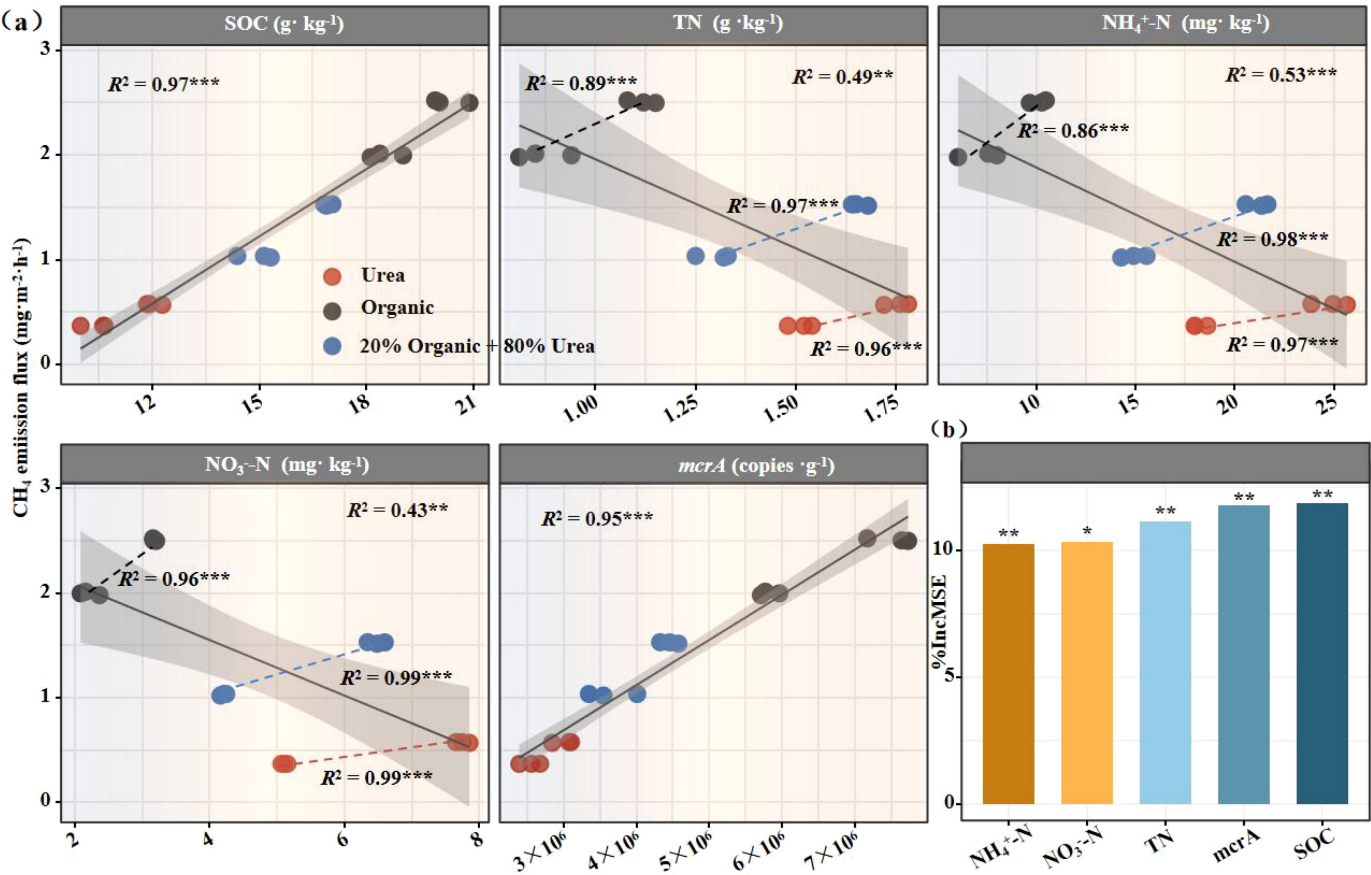
Relationships between CH_4_ fluxes and soil environmental factors. Linear fitting analysis between CH_4_ fluxes and SOC, TN, NH_4_^+^-N, NO_3_^–^N in soil **(a)**; Random Forest analysis evaluating the importance of soil physicochemical properties to CH_4_ fluxes **(b)**. *R* ^2^ represents goodness of fit, and asterisks (*) denote significance levels: * *P* < 0.05, ** *P* < 0.01, *** *P* < 0.001.

To explore the effects of environmental factors on CH_4_ emission fluxes in rice field, multiple statistical analyses were employed to identify the relationships between the soil SOC, TN, NH_4_^+^-N, NO_3_^–^N, *mcrA* gene and CH_4_ emission fluxes (**Figure 5**). In the present study, linear regression analysis demonstrated a significant positive correlation between CH_4_ fluxes and *mcrA* gene copies (*R*² = 0.96, *P* < 0.001) and SOC content (*R*² = 0.97, *P* < 0.001). Conversely, CH_4_ fluxes were negatively correlated with TN (*R*² = 0.49, *P* < 0.01), NH_4_^+^-N (*R*² = 0.53, *P* < 0.001) and NO_3_^–^N (*R*² = 0.43, *P* < 0.01). The above findings indicated that the N fertilizer type exerted a greater influence on CH_4_ emissions than the N application rate.

### 3.6 Impacts of N fertilizers practices on rice yield and quality

**Figure 6:**
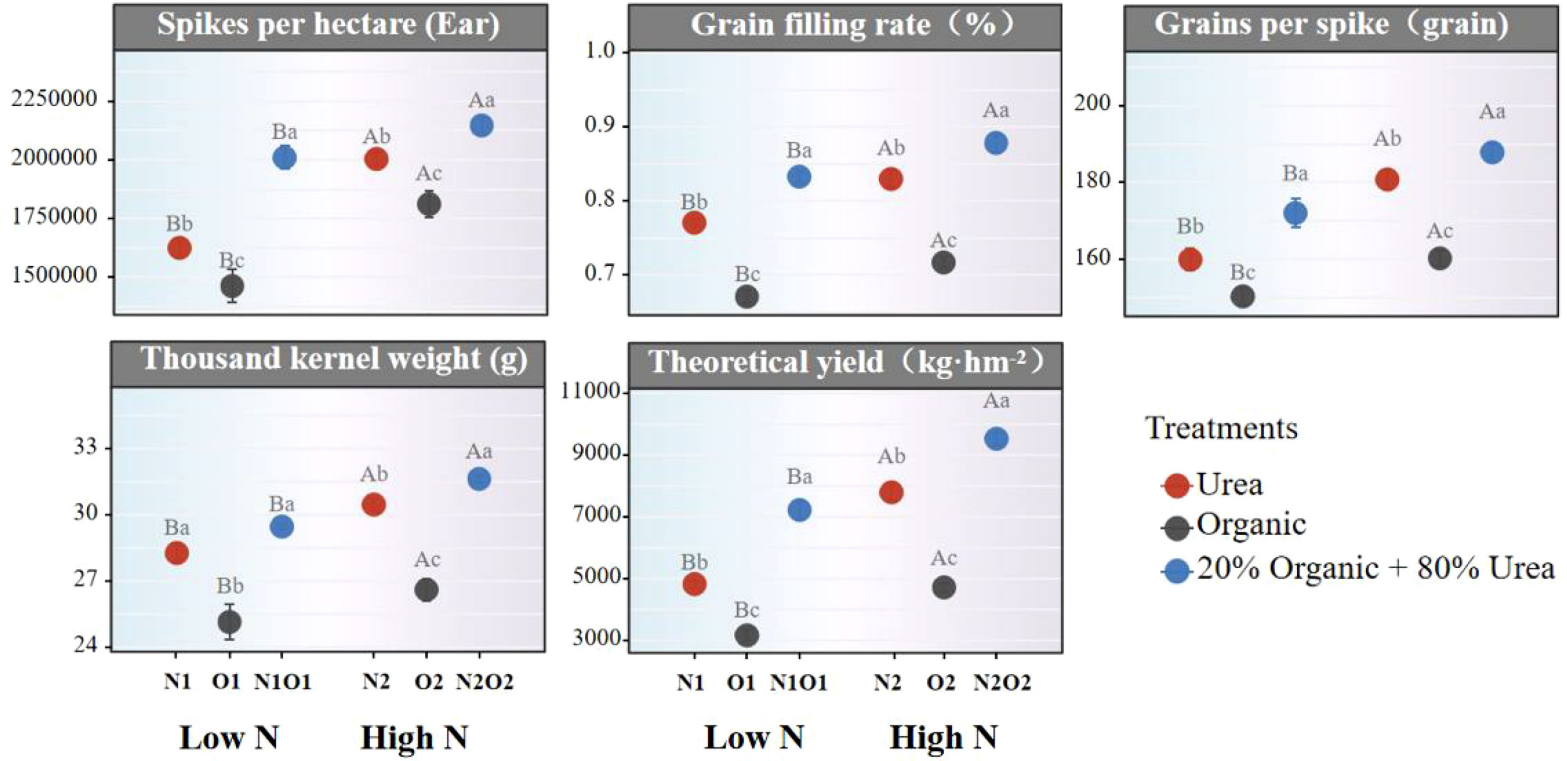
Effects of different treatments on rice yield. Different lowercase letters indicate significant differences (p < 0.05) among treatments under the same nitrogen fertilizer application rate.

To comprehensively evaluate the agronomic performance of different N fertilizers practices, rice yield and quality parameters were analyzed. Results indicated that organic fertilizer combined with urea achieved the highest rice yield across all yield-related metrics (e.g., grain filling rate, thousand grain weight, and theoretical yield) under equivalent nitrogen application rates (**Figure 6**). Conversely, organic fertilization alone yielded the lowest production. Notably, rice yield under low-nitrogen mixed fertilization was comparable to that under high-nitrogen urea fertilization, suggesting the feasibility of maintaining high yields while reducing nitrogen inputs through optimized fertilization strategies.

**Figure 7:**
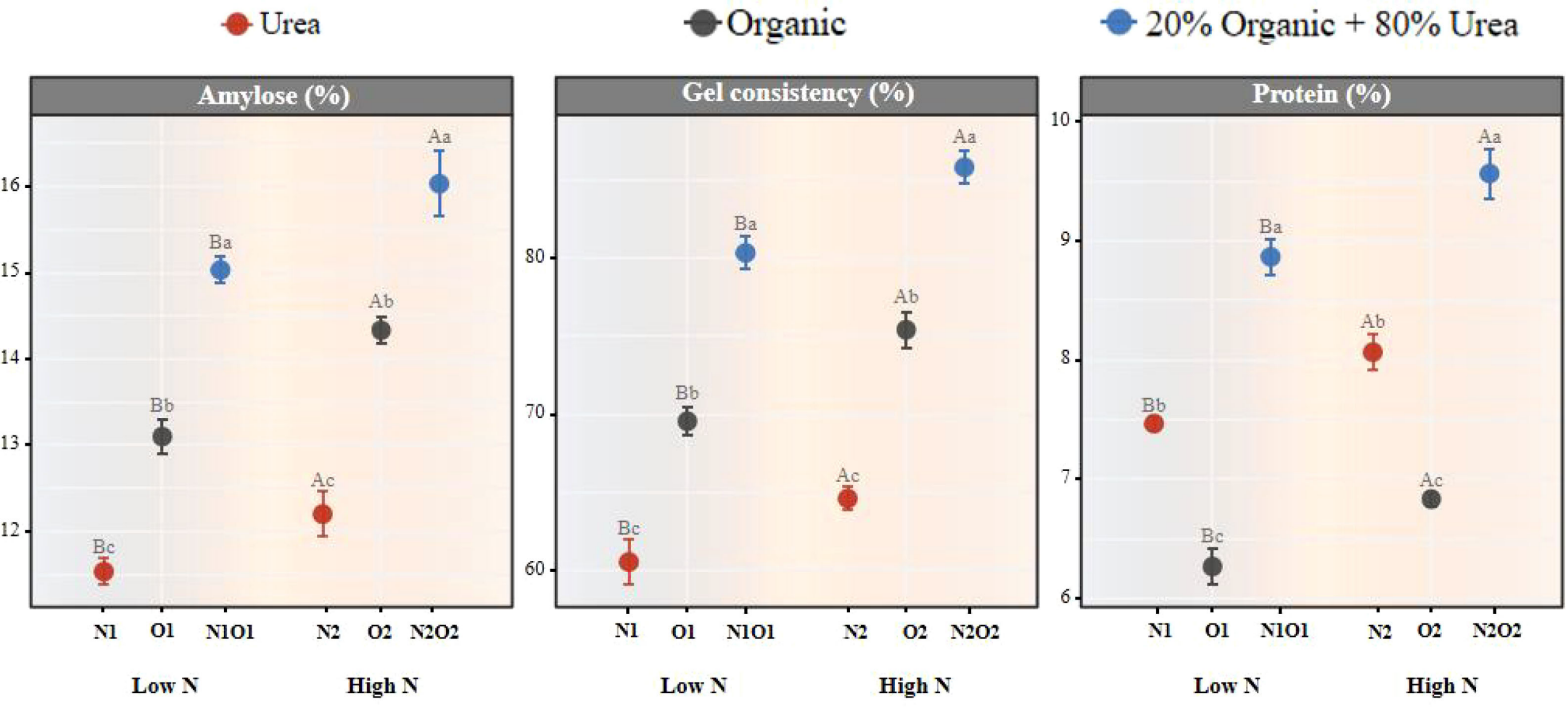
Effects of different treatments on rice quality. Different lowercase letters indicate significant differences (*P* < 0.05) among treatments under the same nitrogen fertilizer application rate.

Regarding grain quality, organic fertilizer combined with urea significantly increased amylose content and gel consistency compared to urea fertilization (*P* < 0.05; **Figure 7**). Protein content was significantly lower under organic fertilization but higher under mixed fertilization. These findings demonstrate that organic-urea mixed fertilization represents the optimal strategy for balancing rice yield and quality.

## 4 Discussion

### 4.1 Effects of different N fertilization management on CH_4_ emissions

N fertilization management practices significantly influenced CH_4_ emissions in rice fields (Liang et al., 2017; Li et al., 2018; Liu et al., 2019). In the present study, CH_4_ emissions exhibited distinct seasonal patterns by N fertilization application and prominent CH_4_ peaks were observed at the stages of heading and tillering, and similar results have been observed in previous N fertilizer applications (Wu et al., 2018; Li et al., 2018; Miao et al., 2020). Such seasonal patterns of CH_4_ emission have been well explained by the fluctuations of soil oxidation reduction conditions regulated by floodwater depth caused by irrigation, and the variations of the activity of methanotrophs and methanogens increased by C and N availability for soil microbes caused by topdressing (Tang et al., 2017; Wu et al., 2018; Liu et al., 2019; Miao et al., 2020; Wu et al., 2020). At the tillering stages, N fertilizer application could accelerate the rapid growth of rice plants and roots, and CH_4_ emission was closely related to rice growth (Kim et al., 2016; Li et al., 2018). CH_4_ emissions in rice fields were highly dependent on rice plant growth (Li et al., 2018), produced CH_4_ is mainly transported by the aerenchyma of rice leaves, sheath and stems (Zhang et al., 2019). Additionally, rice roots provide an organic substrate for methanogens by the exudates or dead root biomass, which stimulates CH_4_ production, and approximately up to 90% of CH_4_ emissions were produced by the above pathways (Jia et al., 2001; Conrad, 2007; Liu et al., 2019; Zhang et al., 2019). Numerous studies have indicated that CH_4_ emission was also strongly affected by soil moisture in rice cropping systems (Wu et al., 2018; Liang et al., 2017; Li et al., 2018). Irrigation practices following N fertilizer application in rice fields can make the soil moisture from the wet to saturated soil conditions, which will provide a better anaerobic environment for methanogenesis, stimulating the enhancement of soil methanogenic bacteria activity, decreasing methanotrophic archaea activity and promoting the production of soil CH_4_ (Li et al., 2018; Liu et al., 2019; Wu et al., 2020). Similarly, in this present study, we also found that CH_4_ emissions were higher at the stages of tillering and heading under irrigation conditions. Compared to the above stages, the relatively lower CH_4_ emissions during the other rice growing periods were likely due to the less pronounced anaerobic conditions under intermittent flooding patterns. Fluctuations O_2_ availability and oxidation reduction conditions in soil regulated by soil moisture caused by irrigation might have shifted the balance between methanotrophic CH_4_ consumption and methanogenic CH_4_ production leading to differences in CH_4_ emission (Fang et al., 2014; Ran, 2016; Liang et al., 2017; Miao et al., 2020; Wu et al., 2020). The previous results of Zhou et al. (2015); Liang et al. (2017) and Wu et al. (2020) confirm the above phenomenon.

Furthermore, levels and types of N fertilizer were also significantly influenced CH_4_ emissions. Similar to the results from other rice cropping ecosystems (Kim et al., 2016; Tang et al., 2017; Liu et al., 2019), increased N fertilizer application rates stimulated CH_4_ emissions by 25.6%-55.7% in our study. Meanwhile, we also found that CH_4_ emission was significantly correlated with soil available N content. Soil NH_4_^+^-N and NO_3_^–^N in urea plots increased by 36.5% and 51.8% respectively, which the results that the copy number of the methanogens gene increased by 18.3%, and stimulated CH_4_ emissions by 55.7%. Increased N fertilizer application rate not only enhanced soil water-filled pore space, with higher soil moisture but also promoted soil mineralization rates and N availability for soil microbes, directly affecting the rates of methanotrophs and methanogens activity to further influence CH_4_ emission (Fang et al., 2014; Yue et al., 2016; Wu et al., 2020). The increased NH_4_^+^-N following N fertilizer application could provide substrate for soil CH_4_ production, increase the availability of NH_4_^+^ to nitrifiers and stimulate the activities of methanogenic archaea and inhibit methanogens activity (King and Schnell, 1994; Wang and Ineson, 2003; Tang et al., 2017; Liu et al., 2019; Wu et al., 2020). Moreover, NH_4_^+^ could interfere with the oxidation of CH_4_, and NH_4_^+^-N oxidation precedes CH_4_ oxidation, because NH_4_^+^ competes with CH_4_ for CH_4_ monooxygenase (Schimel, 2000; Yang et al., 2015), which promotes CH_4_ emission in rice field.

Apart from the application rate of N fertilizer, organic fertilizer application will generally stimulate the CH_4_ emission by 45%-252% in paddy soil (Zou et al., 2003; Tang et al., 2017; Miao et al., 2020). Compared with urea, organic fertilizer application in the experiments of low N and high N significantly increased CH_4_ emissions by 442.1% and 337.3%, respectively, in the present study. The application of organic fertilizer significantly increased soil organic carbon by 68.8% to 77.6%, and increased organic matter can not only effectively provide abundant substrate for soil methanogens, but also consume a large amount of oxygen during the degradation process and formed soil anaerobic environment (Tang et al., 2017), which enhanced copy number of methanogens by 128.6% in O1 plots and 149.9% in O2 plots, respectively. Applied organic fertilizer can not only provide continuous N support for rice plants, but also significantly increase soil temperature and moisture, reduce soil oxygen content, provide better survival environmental conditions and sufficient substrate for methanogens, and promote CH_4_ emission in rice fields at the later stage of rice growth when the paddy soil is relatively dry (Tang et al., 2017; Miao et al., 2020; Jin et al., 2024). However, of special interest is that organic fertilizer combined with urea reduced CH_4_ emissions by 39.2-48.4% compared to organic alone, possibly due to less anaerobic conditions and enhanced methanotroph activity. Soil anaerobic environment is difficult to form in the experiment of organic fertilizer combined with urea, but the above experiment treatment can significantly enhance the activity, diversity and abundance of soil methanogens, resulting in a large amount of CH_4_ being oxidized to CO_2_, and reducing CH_4_ emissions in paddy fields (Zheng et al., 2008; Shao et al., 2022).

### 4.2 Effects of different N fertilization management on rice yield and quality

Organic fertilizer or combination with urea fertilizer has the potential to avoid the environmental damage caused by excessive urea fertilizer application (Kakar et al., 2020). Therefore, it is important to evaluate the impact of organic fertilizer alone or in combination with other fertilizers on yield potential and rice grain quality to secure food supply. In this study, compared with N1, O1 and N1O1, the additional application of N fertilizers significantly increased rice yield by 61.6%, 49.2% and 31.9%, respectively. In addition, rice yield in N1O1 plot was significantly increased by 49.8% and 128.5% compared with N1 and O1 plot, and N2O2 plot increased significantly by 22.3% and 102.1% compared with N2 and O2 (**Figure 6**). Numerous preceding studies demonstrated increasing trends in grain yields with higher N application rates or organic fertilizer combined with urea (Miao et al., 2020; Zhang et al., 2020; Hu et al., 2021, 2022; Jin et al., 2024). Meanwhile, increased N application rates also significantly enhanced the soil available N content such as NH_4_^+^-N and NO_3_^–^N (**Figure 1**). The increased NH_4_^+^-N and NO_3_^–^N in soil can improve effectively the net photosynthetic rate and chlorophyll content, enhance N supplies for the grain filling stage and promoted rapid the development of branches and roots, which beneficial for rapid growth rice plants and improving crop production (Li et al., 2018; Zhang et al., 2019; Tang et al., 2020; Zhang et al., 2020; Li et al., 2023; Jin et al., 2024). Chemical fertilizers and organic fertilizers exhibit different N release characteristics (Peng et al., 2010). Chemical fertilizers such as urea had a higher nutrient release rate in the early stage of application, but caused high nutrient loss that synchronized with the crop nutrient requirement (Peng et al., 2010), resulting in insufficient N supplies during the later stages of crop growth (Jin et al., 2024). On the contrary, organic fertilizer had a lower nutrient release rate during the growing season which may cause insufficient N supply to the grain filling stage of rice (Jin et al., 2024; Song et al., 2024). Organic-urea mixed fertilization can give full play to the advantage of the two fertilizers to ensure adequate nutrient supply during the critical period of rice production (Zhang et al., 2023; Jin et al., 2024). Additionally, productive tillers are responsible and critical elements for rice production which can be affected by the rate or type of application of N fertilizer. Previous studies showed that the combined application of organic and inorganic fertilizers increased tiller number, spikelet number, thousand kernel weight, and yield, which grain yield was controlled by the above components (Kim et al., 2016; Moe et al., 2019; Tang et al., 2020; Zhang et al., 2020; Hu et al., 2021; Li et al., 2023). Consequently, the above explanation strongly indicates that under equivalent N applications, the rice yield of organic fertilizer combined with urea application was significantly higher than that of urea or organic fertilizer alone, which was very consistent with the results of this study.

Similar to grain yield, rice quality (e.g. amylose content, gel consistency, protein, head rice percentage, chalkiness and hot viscosity) was comprehensive traits controlled by the rate and type of N fertilizer application, which the contents of amylose, gel consistency and protein were essential elements to define the grain quality and nutritional value of rice (Ju et al., 2018; Kakar et al., 2020; Tang et al., 2020; Zhang et al., 2020; Zhao et al., 2020; Hu et al., 2021, 2022). Organic fertilizer combined with inorganic fertilizer not only ensured the continuous supply of nutrients at each key growth stage of rice (Zhang et al., 2023; Jin et al., 2024), but also increased the absorption and accumulation of potassium and the transfer of potassium in rice, which improved the appearance and milling quality of rice (Nie et al., 2016; Li et al., 2023). More importantly, the addition of organic fertilizer could increase the chlorophyll content, improve the photosynthetic rate of rice plants, promote the generation of photosynthetic products and the efficiency of transport to rice grains, which nutrients were fully gathered in rice grains, and thus improve rice quality (Li et al., 2023). In the present study, compared with urea or organic fertilizer alone, organic fertilizer combined with urea significantly increased amylose content, gel consistency, and protein (**Figure 7**), which is consistent with the results of Gao et al. (2024). Together, these results suggest that organic-urea mixed fertilization is an effective method both for rice yield production and quality.

### 4.3 Limitations

In the present study, although optimizing organic fertilizer combined with urea practices enhanced rice grain yield, improved rice quality and mitigated CH_4_ emissions, the impacts of optimizing organic fertilizer combined with urea on the microbial activity of methanogens and methanotrophs at the species or genus scale have not been explicitly addressed, in particular, abundance and diversity of the above functional genes. Additionally, the application of organic fertilizer changed soil water-filled pore space, reduced soil oxygen content, and increased soil moisture and pH, which significantly stimulated CH_4_ emissions, especially in the fallow stage of rice fields. Therefore, the lack of monitoring of CH_4_ emissions from organic fertilizer combined with urea experiments during the non-growing season seriously affects the estimation of greenhouse gas inventories in rice-cropping ecosystems. Targeted research is needed to clarify the microbial activity of methanogens and methanotrophs to reveal the molecular biological mechanism of CH_4_ production, and evaluate CH_4_ emissions budgets during the growing season and the fallow stage of rice fields under organic fertilizer combined with urea practices in rice-cropping systems.

## 5 Conclusion

In summary, the CH_4_ emissions, rice grain yield and quality were comprehensively controlled by the rate and type of N fertilizer application during the rice growing season. The CH_4_ emissions in paddy soils significantly increased with increasing N fertilization. Compared with urea, organic fertilizer application significantly increased CH_4_ emissions, while organic fertilizer combined with urea significantly decreased CH_4_ emissions relative to organic fertilizer. In addition, we also found that organic fertilizer combined with urea significantly increased rice grain yield, amylose content, gel consistency and protein content. Therefore, Therefore, optimizing organic and urea combinations offers a sustainable strategy for subtropical rice systems, enhancing yield and quality while mitigating CH_4_ emissions.

## Data Availability

The original contributions presented in the study are included in the article/supplementary material. Further inquiries can be directed to the corresponding author.
